# eDNA metabarcoding of small plankton samples to detect fish larvae and their preys from Atlantic and Pacific waters

**DOI:** 10.1038/s41598-021-86731-z

**Published:** 2021-03-31

**Authors:** Eva Garcia-Vazquez, Oriane Georges, Sara Fernandez, Alba Ardura

**Affiliations:** 1 Department of Functional Biology, University of Oviedo, C/Julian Claveria S/N, 33006 Oviedo, Spain; 2 Department of Natural Sciences, School of Science and Computing, Marine and Freshwater Research Centre, Galway-Mayo Institute of Technology, Dublin Road, Galway, H91 T8NW Ireland

**Keywords:** Computational biology and bioinformatics, Ecology, Molecular biology, Environmental impact

## Abstract

Zooplankton community inventories are the basis of fisheries management for containing fish larvae and their preys; however, the visual identification of early-stage larvae (the “missing biomass”) is difficult and laborious. Here, eDNA metabarcoding was employed to detect zooplankton species of interest for fisheries from open and coastal waters. High-Throughput sequencing (HTS) from environmental samples using small water volumes has been proposed to detect species of interest whose DNA is the most abundant. We analyzed 6-L water samples taken from subtropical and tropical waters using Cytochrome oxidase I (COI) gene as metabarcode. In the open ocean, several commercial fish larvae and invertebrate species important in fish diet were found from metabarcodes and confirmed from individual barcoding. Comparing Atlantic, Mediterranean, Red Sea, and Pacific samples we found a lower taxonomic depth of OTU assignments in samples from tropical waters than in those from temperate ones, suggesting large gaps in reference databases for those areas; thus a higher effort of zooplankton barcoding in tropical oceans is highly recommended. This and similar simplified sampling protocols could be applied in early detection of species important for fisheries.

## Introduction

The study of plankton communities is essential for fisheries. Planktonic larvae stages are present in the many commercial fish species life cycle, but they are identified as “missing biomass”, because most of them are still unknown^1^. Moreover, they are an important part of the trophic chain being food for other and, sometimes the same, plankton species^2,3^. These planktonic communities are especially sensitive to climate change and other environmental alterations^4^, being potentially vulnerable to losses of species diversity^5,6^, likely related to alterations in the trophic chain and, in particular, in their preys. This is the case of copepods^5^, which are food for larvae of important commercial species, such as hakes^7^. Previous studies show that copepod diversity presents a latitudinal cline associated with temperature^8^, with evidences of zooplankton decline in tropical Atlantic waters^9^, and this can put at risk the sustainability of fisheries. Therefore, large-scale studies on ichthyoplankton communities are necessary, especially on their main preys at tropical latitudes.

However, the study of plankton communities using traditional tools is difficult. The filtration of large volumes of water is necessary for concentrating the plankton, identifying the individuals visually under the microscope one per one, and doing the taxonomical classification. Besides, the complexity of zooplankton communities, with high number of cryptic species and the presence of larval stages difficult to identify, make it laborious to identify species and assess biogeographic patterns from local to global distribution with traditional sampling methods^10^. Ardura et al.^11^ have shown that DNA Barcoding is a promising methodology for ichthyoplankton inventory, and Fuentes et al.^12^ have applied quantitative Polymerase Chain Reaction (qPCR) for quantifying toxic algae, but individualization is still necessary, and new developments are still needed for application in large-scale routine surveys. In the past decade, there has been a growing interest in the use of high throughput sequencing (HTS) techniques that obtain millions of DNA sequences from water samples (environmental DNA or eDNA) representing the biota inhabiting those waters. The new methods of HTS on environmental samples, generically called metabarcoding, can help in species detection and inventory, as has been proven for Polarstern ballast water^13–15^, open waters^16^, estuaries^17^, marine benthos^18^ and biofouling communities^19^, among others. They are employed for prokaryotes and some specific taxonomic groups like ciliates, in large oceanic expeditions like *Tara Oceans*^20,21^, but have been less applied for whole eukaryotic communities, and very little to target zooplankton fish prey, to our knowledge.

eDNA metabarcoding is an interdisciplinary method that brings together traditional field-based ecology with in-depth molecular methods and advanced computational tools^10^. This has enormous advantages in the study of biodiversity, because the sampling methodology is easy and non-destructive. Disturbing biota to be examined is not necessary, since they will be identified from DNA molecules they shed in the environment. The methodology based on metabarcoding is being used for the rapid detection of new coming species, studies about food webs, monitoring the ecosystem health and impacts of the global change^22^. However, as any emerging science, some pitfalls have been considered. One of the most important is that every step of the methodology influences the final results, from sampling and DNA extraction to bioinformatics analysis, including the design of optimal primers to have minimal bias with a good resolution^22^. Being the standardization of the process very important^23,24^, if appropriate controls are included along the data generation process, DNA metabarcoding will provide a valuable tool in ecosystem assessments^25^.

Metabarcoding is based on the comparison of unknown sequences with references; thus, its efficiency for plankton monitoring relies on the accuracy and completeness of reference databases. Bucklin et al.^22^ and Weigand et al.^26^, amongst other authors, pointed that the accuracy of species identification requires reliable reference sequence libraries of known taxa. The abundance of references determines in practice the marker to be used as a metabarcode^27^. The DNA barcode of choice employed in the first marine barcoding projects was the cytochrome oxidase I gene (COI)^28,29^. While it is true that using primers for metabarcoding can lead to biases if these primers do not perform equally in all the organisms, previous studies have shown that almost the entire aquatic macroinvertebrate community can be reliably detected with COI metabarcoding, using computational evaluations as well as experimental data^30^. The gene coding for the ribosomal 18S RNA is another barcode of increasing importance in metabarcoding projects, being most used for the assessment of phytoplankton community and food diet characterization^31,32^. However, the phylogenetic resolution of 18S RNA is smaller than that of COI, being only comparable at phylum level, since taxa cannot be clearly identified at species level^33^. On the other hand, the taxonomic coverage of these genes in databases is unbalanced, being wider for COI in aquatic invertebrates, as reported by Ardura^27^.

Whatever the marker employed, reliable and quick bioassessments are of critical importance for biomonitoring and environmental impact assessment of aquatic ecosystems, and DNA metabarcoding has the potential to meet this challenge, detecting species with low density in a fast, and relatively cheap, way. Even relatively scarce species can be successfully detected from small water volumes—as small as 3 L, see for example inventories of port species in Borrell et al.^34^. Metabarcoding on small water samples could therefore serve for early alert of adverse phenomena like non-indigenous biological invasions^34^, or potentially toxic red tides^35^, because causative species can be detected from such minimal volumes of water. These examples correspond to coastal waters, where zooplankton density can be as high as more than 50,000 individuals/m^3^ (for example in Manila Bay; Jose et al.^36^); moreover, similar densities can be found even for single species in some African estuaries^37^. Thus, application of HTS on small water samples seems to be suitable for estimating biodiversity in these habitats.

However, the application of protocols based on small volumes of water may be questionable in the open ocean where the plankton density is much lower, like for example 400–1200 individuals m^−3^ in Alboran Sea^38^, or less than 500 off West African coasts, with higher values in the plume of some estuaries^39^. Although reported densities of zooplankton in open waters vary enormously depending on depth, nutrients, oxygen, and season (in the open Mediterranean Sea, zooplankton density can oscillate between 34.5 to as little as 0.8 mg of biomass m^−3^, see a review in Siokou-Frangou et al.^40^), they tend to be higher in the vicinity of the coast in all the oceans. Another example is Osaka Bay to Kii Channel (Japan, Pacific Ocean), where zooplankton biomass decreases from more than 20 inside the bay down to less than 1 mg m^−3^ in open waters, with large differences between oligotrophic and eutrophic zones^41^.

Given generally low densities in the open ocean, zooplankton species to be detected from a few liters of high seas water are likely the most abundant or dominant ones, being thus able to locate zones rich in fish larvae and their preys. In this proof of concept, we will look for that “missing biomass” using metabarcoding with the COI marker in water samples of small volume (a few liters), from coastal waters and open sea. Expectation was to detect more species richness from coastal samples. Results will help to tackle the use of this type of easy-sampling protocols for monitoring zooplankton communities and detecting missing biomass important for fisheries.

## Materials and methods

### Study areas

**Figure 1:**
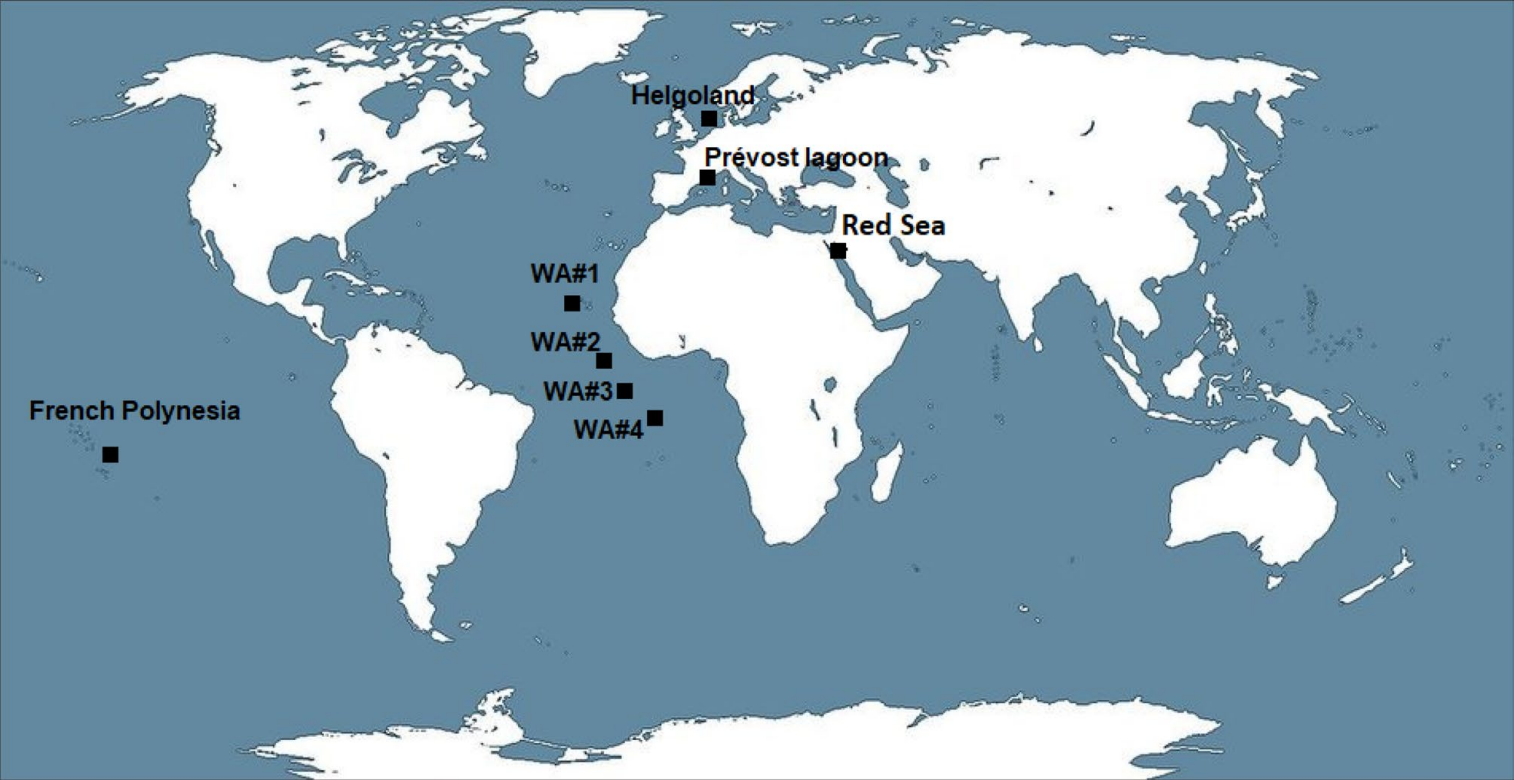
Map with the five sampling points. West African waters: WA1, WA2, WA3 and WA4. Helgoland: in front of Bremerhaven port-North Sea. Prévost Lagoon-Mediterranean Sea. Red Sea-Gulf of Aqaba. French Polynesia-Rangiroa atoll. Map obtained from www.wikimedia.org, with Creative Commons License, and modified with Paint software 2004.

Sampling sites were chosen in order to compare the DNA metabarcoding monitoring obtained from open versus coastal waters, and subtropical versus tropical waters. Therefore, water samples were taken from four zones in the open subtropical (WA #1) and tropical Atlantic Ocean (WA #2–WA #4) (Fig. 1), and from four zones near the coast: in the North Sea (offshore from Bremerhaven port; October 2016), the Mediterranean Sea (inshore, within Prévost lagoon; December 2016), the Red Sea (inshore, in the Gulf of Aqaba, between Red Rock and Neviot beaches, January 2019), and the tropical Pacific Ocean (inshore, inside Rangiroa atoll, French Polynesia; February 2018) (Fig. 1). The currents that affect these sampling points and other habitat characteristics are in Table 1. From chlorophyll data at the time of sampling (NASA Earth Observatory, 2020, https://earthobservatory.nasa.gov/global-maps/MY1DMM_CHLORA), three of the open ocean samples taken off West Africa were within oligotrophic minimum oxygen zone.

**Table 1:** Water samples analyzed (6 L). Taxonomic diversity as Brillouin D-index calculated from the number of species by class.

Sample name	Polynesia-C	NorthSea-C	Med-C	RedSea-C	West Africa #1	West Africa #2	West Africa #3	West Africa #4
Site	Rangiroa atoll, French Polynesia	NW of Helgoland, North Sea	Prévost lagoon, Montpellier, Mediterranean	South of Israel, Gulf of Aqaba	W of Nuakchot, East Atlantic	SW of Liberia. East Atlantic	NE of Ascension Island, East Atlantic	E of Ascension Island, East Atlantic
Latitude	− 15.12	54.2	43.61	29.32	18.63	− 2.43	− 5.12	− 7.49
Longitude	− 147.63	7.46	3.88	34.57	− 23.89	− 10.48	− 8.58	− 6.51
Main current	Pacific South Equatorial	Jutland	Ligurian-Provence	Tidal currents	Canary	Atlantic Equatorial Counter	Guinea and Atlantic South Equatorial	North Benguela
Chlorophyll	Oligotrophic	Mesotrophic	Mesotrophic	Meso-Eutrophic	Mesotrophic	Oligotrophic	Oligotrophic	Oligotrophic
Location	Inshore	Offshore	Inshore	Inshore	Offshore	Offshore	Offshore	Offshore
Replicates	2 (3L)	3 (2L)	3 (2L)	6 (1L)	1	1	1	1
Total zooplankton sequences	6294	11,172	14,794	13,335	5343	13,937	5581	22,649
Taxonomic diversity	1.262	1.462	1.376	0.808	0.347	0	0.597	0.597

### Sampling methodology

In the main study, we have analyzed water samples of 6 L per sampling point. Samples from the open ocean were obtained onboard RV Polarstern during the research cruise PS116 in November 2018^42^. Polarstern has an underway system to pump water from outside the vessel into the laboratories. The tap connecting with the membrane pump, with the water take at 6 m of depth, was running constantly while underway. Each sampling day, ca. 6 L of water were concentrated through 0.2 μm pore filters, coded and stored in absolute ethanol for further DNA extraction and analysis. To confirm the species found from metabarcoding with an independent method, individual zooplankton samples were taken overboard with a manual plankton net of 20 μm mesh, 200 L concentrated in three replicates of 50 mL. Samples were filtered through a 0.2 μm membrane. Zooplankton individuals were inspected under the microscope, taxonomically identified with the use of zooplankton guides, a few were transferred to ethanol for subsequent DNA barcoding, the rest were fixed in 10% formaldehyde.

Along the Mediterranean coast at Prévost lagoon, 6 L of surface water were collected in three sterile bottles of 2 L (three subsamples) and filtered through 0.2 μm polyethersulfone filters. Samples from Rangiroa (French Polynesia) were two 3 L water subsamples were collected in separate sterile bottles and filtered immediately in situ with a syringe system with a Swinnex portafilters from Merck Millipore and a membrane filter with 0.2 µm pore size and 25 mm diameter, and filters were stored in ethanol until eDNA extraction. Samples from the Red Sea (Gulf of Aqaba) were taken from the shore using also a syringe system and one membrane per liter of water. The water was filtered on field using PES Supor 200 Membrane Filters (Pall Corporation, Life Sciences) with 0.2 μm pore size and 47 mm diameter. The filters were preserved in 100% ethanol at room temperature.

North Sea samples of water (three 2 L samples) were taken from RV Polarstern during expedition PS102 after loading ballast water in Bremerhaven port. Samples were vacuum filtered through 0.2 µm Nucleopore™ membranes that were immediately stored in ethanol.

A second study was done with smaller water volumes. On the same PS102 cruise, in November 2016, we obtained water samples overboard with a bucket, and filtered 1.5 L through 0.2 μm mesh membranes to be stored in ethanol. These samples were taken from latitude 48.49 N in the North Sea to latitude 22.22 S (Supplementary Table 1).

In all cases, we used sterile equipment (gloves, syringe, bucket) that was carefully cleaned before and after sampling with bleach diluted at 10% (5% sodium hypochlorite) and triple-rinsed with clean sterile water.

### HTS procedure

DNA was extracted in all cases from filters using PowerWater DNA Isolation Kit (Qiagen) following manufacturer’s instructions. A previous step to pellet the ethanol content and include it in the extraction process was included to avoid starting material loss. All extractions were performed in a per-PCR laboratory under a flow laminar hood equipped with UV light. Negative controls were employed during extraction. For the Polarstern samples, a fragment from the *COI* gene was amplified with polymerase chain reaction (PCR) from the extracted eDNA using the universal primers mlCOIintF^43^ and jgHCO2198^44^ that were modified with a PGM sequencing adaptor, the barcodes (one per sample) needed to differentiate the reads belonging to each water sample, and a “GAT” spacer. Amplification was carried out in a total volume of 20 μL including Green GoTaq Buffer 1×, 2.5 mM MgCl2, 0.25 mM dNTPs, 20 pmol of each primer, 4 μL of template DNA, 200 ng μL^−1^ of bovine serum albumin, and 0.65 U of DNA Taq polymerase (Promega). PCR conditions in the Veriti Thermal Cycler (Applied Biosystems, Foster City, California) were 95 °C for 1 min, followed by 35 cycles of 95 °C for 15 s, 46 °C for 15 s, 72 °C for 10 s, and a final extension of 72 °C for 3 min. Extraction (N = 3) and field (N = 1) negative controls were included in the PCRs. The amplification success was visually assessed on 2% agarose gel. PCR amplicons were purified from agarose gel using the Montage DNA Gel Extraction Kit (Millipore); quantified using the Qubit BR dsDNA Kit (Thermo Fisher Scientific); and double‐checked in a Bioanalyzer 2100 (Agilent Technologies) to confirm the fragment size, the absence of by‐products, and to do a more precise quantification^45^. For the rest of the samples, the same metabarcoding region was amplified, but using an Illumina approach as described in Ardura et al.^31^. Summarizing, the primers were modified to include IlluminaTM overhang adaptors and sample-specific indices following the dual-PCR Illumina protocol (https://support.illumina.com/), where the conditions for the amplicon PCR were changed to the ones described by Leray et al.^43^. In addition, bovine serum albumin (BSA) was added to the PCR reactions to increase PCR yields from low purity templates and to avoid, as much as possible, the effect of inhibitors presents in the water. After library construction, MiSeq Illumina platform was employed to run the sequencing step using paired-end sequencing (2 × 301).

PCR replicates were not developed, but sampling replicates were taken when possible (Table 1), in order to recover as much of the biodiversity as possible.

### Individual barcoding

DNA was extracted using silica gel columns [QIAmp DNA Mini Kit, DNeasy Blood and Tissue kit (Qiagen)]. Aliquots were frozen at – 20 °C for long-time preservation, even for years.

The mitochondrial COI gene was amplified using the universal primers designed by Leray et al.^43^ as in the metabarcoding analysis. An extra 400–600 bp fragment within the nuclear small subunit ribosomal DNA (18S rRNA gene, 18S thereafter) was amplified by PCR with Uni18SF and Uni18SR primers and protocol described by Zhan et al.^45^.

PCR products were visualized in 2% agarose gel dyed with SimplySafe (EURx Ltd). Purification and sequencing were performed in Sequencing Unit of the University of Oviedo (Spain). Sequences were BLASTed against GenBank database within NCBI (https://blast.ncbi.nlm.nih.gov) using best match for species assignment, > 97% identity for *COI* and > 80% for 18S^46^. When no genetic identification was possible with the COI gene, the reference for 18S rRNA gene was considered. Taxonomic information was checked in World Register of Marine Species (WORMS; http://www.marinespecies.org/).

### Bioinformatics analysis of metabarcoding data

Adaptors from sequencing platforms were trimmed within the platform software and fastq sequences files were used to filter per quality. Qiime2 2020.2^47^ was used to trim the primers and to filter by length (amplicon size 200–400 bp reads were retained) using Cutadapt 1.15^48^. Denoising option was employed where sequences are dereplicated and denoised using the unoise3 algorithm from Usearch 11.0.667_i86^49^. For taxonomic classification, filtered sequences were compared against a public COI reference database (NCBI, accessed on 16/06/2020) and stored locally. The database was downloaded using the esearch query “COI NOT Bacteria NOT environmental NOT viruses NOT unclassified" and constructed with the respective taxonomic information using the script “Entrez_qiime.py” by Baker^50^. Finally, “qiime feature-classifier” command was employed to assign the taxonomy, using a 97% as identity percentage and an e-value of 10^–50^.

Resulting Operational Taxonomic Units (OTUs) from taxonomic assignation were employed in further steps.

### Data analysis

First, the OTU tables were inspected manually and non-marine species (e.g. human, insects, etc.) were eliminated. Analyses were done only on species that, from their biological cycle, could be present in plankton in the sampling area at the sampling time. The analyses described further were made only on zooplankton.

Diversity was estimated with the Brillouin D-index^51^, which is preferred over the Shannon index when the species differ in their capture rates. This is the case of PCR-based metabarcoding, because, as explained above, not all the taxa are detected with the same probability or taxonomic depth.

Tridimensional, non-metric multidimensional scaling (NMDS) was employed to visualize differences between samples. Clustering analysis of the samples was made employing UPGMA and 1000 bootstraps. These multidimensional analyses were based on Gower distances calculated from presence (1) or absence (0) of species DNA.

Mann–Whitney test was employed to compare groups of samples (e.g. temperate/subtropical versus tropical samples) for the taxonomic precision of the assignment (i.e. percentage of OTUs assigned to species). Monte Carlo (9999 permutations) was also employed to assess errors. Statistics was done with the free software PAST^52^.

## Results

Overview of HTS data and taxonomic assignment depth HTS results are summarized in Supplementary Table 2, including the GenBank accession numbers of sequences. The list of OTUs found in all the samples and their taxonomic assignments is in Supplementary Table 3.

In the samples of 1.5 L obtained from PS 102 Polarstern after quality filtering, only the sample taken near the coast yielded sequences taxonomically assigned to marine invertebrates, all found in European waters: four Polychaetae (*Gyptis propinqua*, *Lumbrineris latreilli*, *Paradoneis ilvana*, *Polycirrus* sp.) and one Malacostraca (*Pisidia longicornis*), with a total of 457 reads. In the other seven DNA samples, marine animals were not detected. Only a few reads of *Homo sapiens* and insects were found, that can be explained from contamination. Thus, small water volumes, such as 1.5 L, do not seem useful to capture zooplankton diversity in open waters from metabarcoding using COI as a metabarcode.

**Figure 2:**
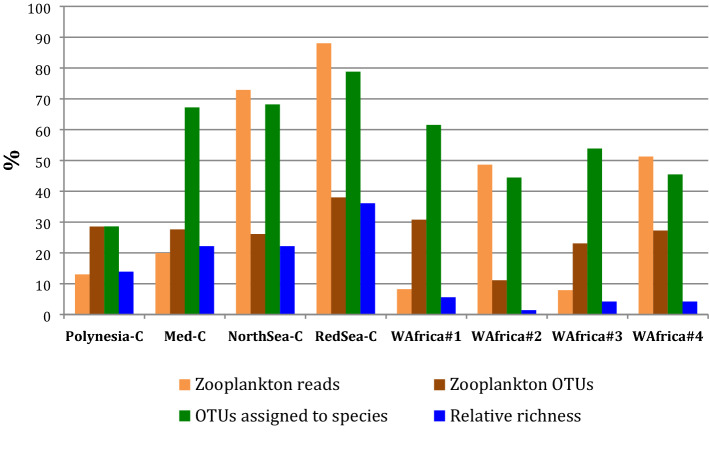
Overview of NGS metabarcoding results in the samples analyzed, as: percent of zooplankton reads or OTUs, and percent of OTUs assigned to a species level in each sample. Relative richness: % of species in a sample over the total number of species detected from metabarcoding in this study.

In the samples of 6 L, the number of quality reads was not different between sampling sites, with sequences of COI metabarcode taxonomically assigned to zooplankton taxa oscillating between 5343 (West Africa #1) and 22,649 (West Africa #4). Samples obtained from coastal areas did not detect more zooplankton sequences than samples from the open seas (Table 1) and ranged from 6294 in Rangiroa (Polynesia) to 14,794 in the Mediterranean Prévost lagoon. The proportion of sequences assigned to animal species that are planktonic, at least in a life stage (percentage of zooplankton in all the assigned reads), was quite different among samples, but the proportion of zooplankton OTUs was quite similar, between 20 and 40%, except in the sample West Africa #2 with about 10% zooplankton OTUs (Fig. 2). The rest of assigned species were principally large animals in the West African open seas, and phytoplankton in the three coastal samples (Supplementary Table 3).

The depth of taxonomic assignments was greater in the four samples from temperate-subtropical waters with more than 60% OTUs assigned to species (North Sea, Red Sea, Mediterranean lagoon, and West Africa #1 that is in Canary Islands) than in the four samples from tropical waters. In three of the tropical samples, more than one half of OTUs were assigned at genus, family, or even order (Fig. 2, Supplementary Table 3). The difference between the two groups of samples was significant (Mann–Whitney U of 0, z = 2.165 with *p* = 0.03; Monte Carlo permutation test with *p* = 0.028).

### Zooplankton identified and its importance for fisheries

#### Open West African waters

Among zooplankton species identified from DNA metabarcoding in West African open waters (Table 2) were three copepods (*Clausocalanus furcatus*, *Delibus nudus*, *Paracalanus nanus*), one Cladoceran (*Evadne cf. spinifera*), one Euphasiidae (krill *Euphausia* sp.), one bivalve (*Magallana gigas*), and one fish (*Engraulis encrasicolus*). In addition, we found DNA of other vertebrates that cannot be part of the zooplankton in any stage, and a few phytoplankton OTUs (Supplementary Table 3). The number of zooplankton species found from each sampling zone was small (see Table 2 and Relative richness in Fig. 2): only one in West Africa #2, three in West Africa #3 and #4, and four in West Africa #1. Six of these species are abundant and typical of zooplankton in the studied areas (Table 2).

**Table 2:**
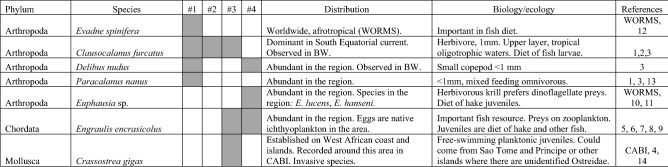
Biological and ecological traits of zooplankton species found from NGS metabarcoding in West African high seas. Presence of species in each sampling zone are marked with gray squares.

All the zooplankton species found from metabarcoding could be identified by eye, or at least individuals morphologically similar to them, including spat of bivalve similar to *Magallana gigas*, and fish eggs similar to anchovies. Although individual barcodes could not be obtained from all of species due to poor DNA extraction, likely due to the fixation of specimens, *Euphasia* and *Delibus* were confirmed from independent COI barcodes (GenBank accession numbers: MW217272 and MW217273, respectively). Other organisms identified by sight from those areas and confirmed with COI and/or 18S rRNA, were the Appendicularia *Oikopleura* sp., larvae of the echinoderm *Styracaster paucispinus*, and the polychaetae *Vanadis formosa* (GenBank accession numbers: MW217563, MW217564 and MW217565, respectively). These species were undetected with metabarcoding, which was expected given the enormous difference of water volume that had to be filtered to obtain individuals for visual examination (200 L) and to do metabarcoding (6 L).

Five of these species identified from 6 L water in open ocean were important for fisheries (Table 2). *Paracalanus nanus* and *Euphausia* are part of hake juveniles´ diet, and *Evadne spinifera* and *Clausocalanus furcatus* are important in the diet of several fishes. *Engraulis encrasicolus*, the Atlantic anchovy, is an important fish resource itself, and also a prey of hake tuna and other fish commercially important for African countries.

#### Coastal waters

Expectedly, in coastal samples the number of zooplankton species’ DNA found from similar water volumes was clearly larger than in open waters, despite similar numbers of reads (see Relative richness in Fig. 2, and Table 3): 26 OTUs in the Red Sea, 16 in the North Sea and the Mediterranean lagoon, and 10 in Rangiroa. Diverse higher taxonomic groups were found: six Phyla from the North Sea and Polynesian samples, five from the Mediterranean lagoon, and four from the Red Sea (Table 3). Some of the species found from metabarcoding could be confirmed from individual barcodes (GenBank accession numbers KT988324 *Mytilus galloprovincialis*, MK295019 *Paracalanus parvus*, MK295022 *Undinula vulgaris*, MK295025 *Acartia clausii*).

**Table 3:**
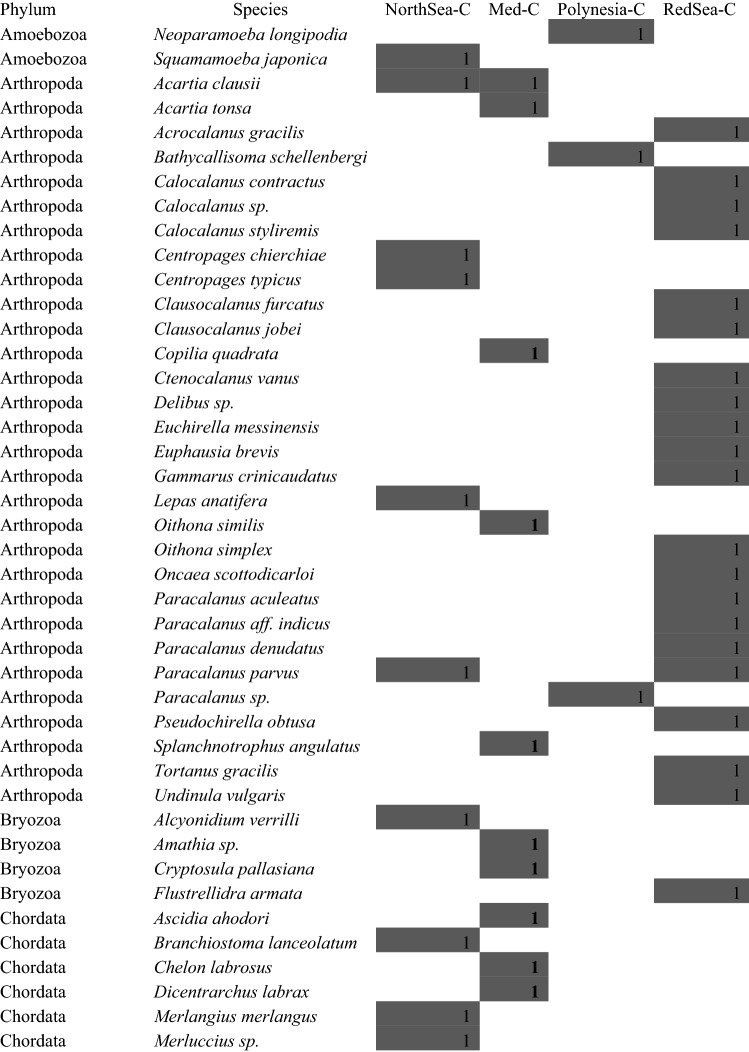

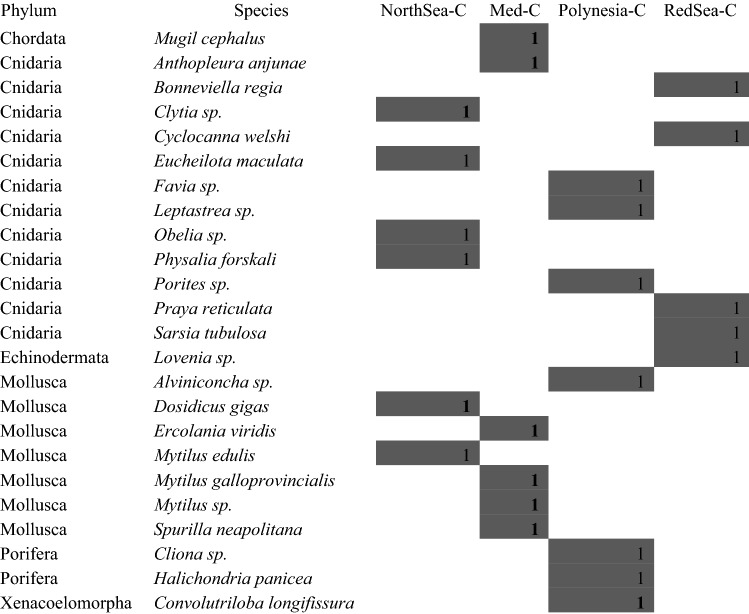
Species identified from COI metabarcoding in the four coastal locations (C) examined in this study. Presence of species in each sampling zone are marked with gray squares.

As in open water samples, several species were commercially important fish whose eggs and early larvae are part of zooplankton, such as *Merlangius merlangus* and *Merluccius merluccius* in the North Sea or *Chelon labrosus* and *Dicentrarchus labrax* in the Mediterranean (Table 3). Some copepods important for fish diet, like *Clausocalanus, Paracalanus* and others*,* were also found.

### Taxonomic profiles

**Figure 3:**
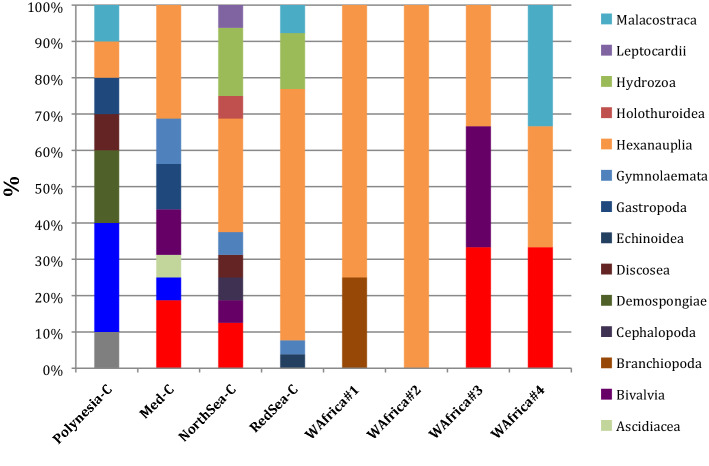
Taxonomic diversity of zooplankton in the analyzed samples, as proportion of species in different classes detected from metabarcoding.

Despite minimal water volumes, considerable taxonomic diversity was captured in coastal samples (Fig. 3), where many different classes of zooplankton were found. In open-sea samples, only DNA of abundant species was detected, as seen above, and consequently diversity was much lower there (see Brillouin taxonomic diversity in Table 1).

**Figure 4:**
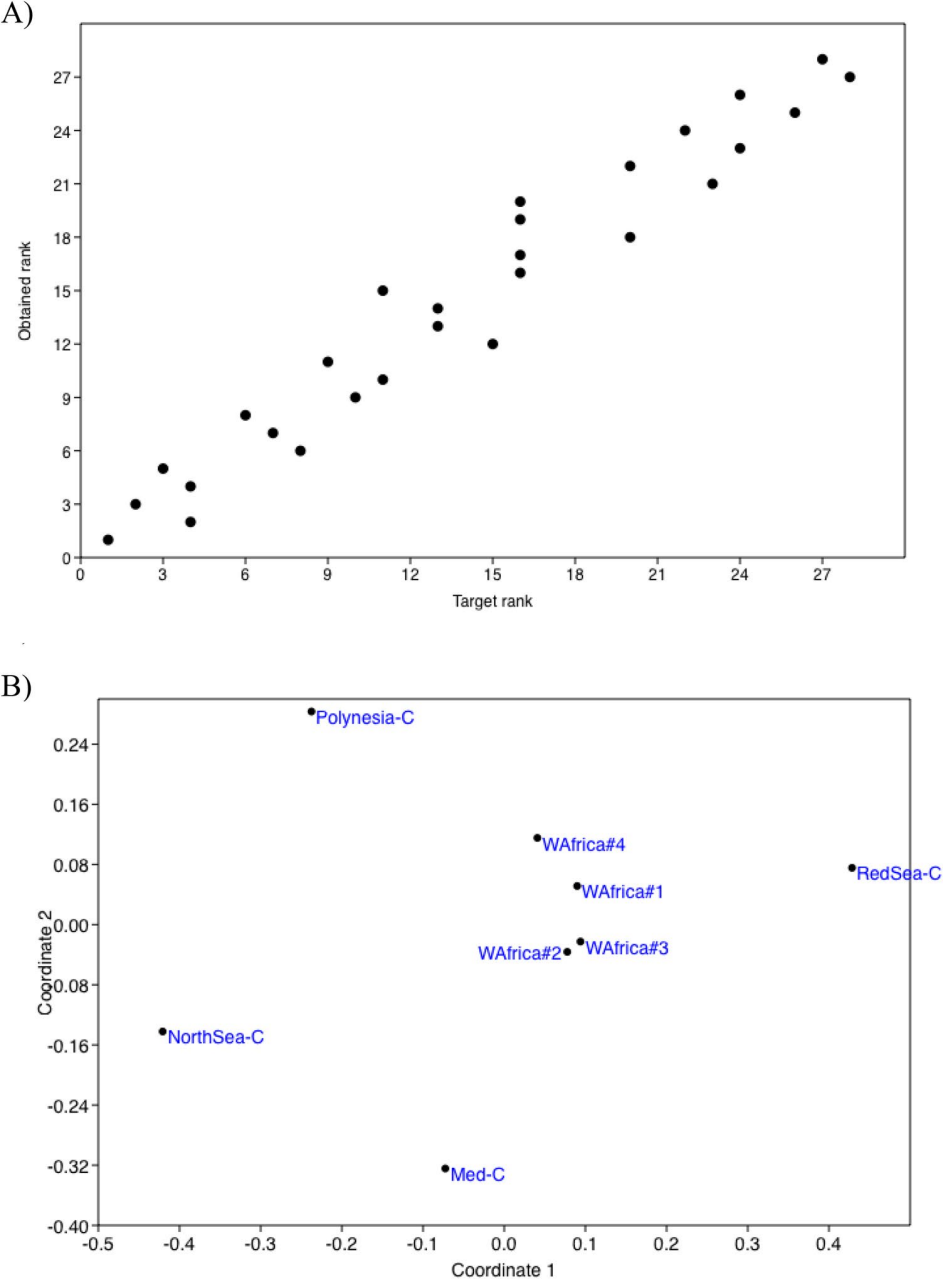
Shepard (**A**) and scatter plot (**B**) of NMDS constructed from presence (1)/absence (0) of species detected from DNA metabarcoding in the samples analyzed.

Indeed, the samples were different to each other, as expected from their distant origins. According to the species found from metabarcodes, the NMDS had an acceptable stress of 0.11, r^2^ of axis 1 of 0.451 and of axis 2 of 0.314 (see Shepard plot in Fig. 4A). Although the four open-sea samples were located close to each other, with WAfrica#2 and WAfrica#3 closer than the other two (Fig. 4B), they all had different zooplankton composition (Table 3).

**Figure 5:**
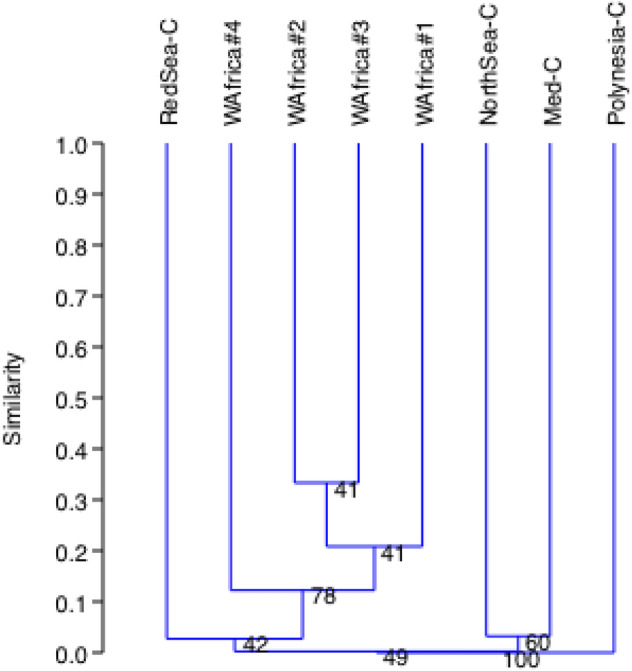
Clustering analysis of sample diversity. UPGMA based on Bray–Curtis distance matrices constructed from presence (1)/absence (0) of the species detected from DNA metabarcoding (10,000 bootstraps).

A UPGMA tree showed clusters that were generally consistent with the geographical locations of the samples. West Africa #2 and #3 samples clustered together, then West Africa #1 and, in a different branch with 78% bootstrap, West Africa #4 appeared separately but in the same clade (Fig. 5). Then, the Red Sea sample joined the cluster with low bootstrap. Another cluster contained the two European coastal samples (NorthSea-C, off Bremerhaven; Med-C, Mediterranean lagoon), connected with the Red Sea sample also with low bootstrap, which is expected because the Mediterranean and Red seas are connected through the Suez Canal. The coastal sample from the tropical Polynesian was in an independent branch clearly separated (100% bootstrap).

## Discussion

The results obtained in this study demonstrate the potential of eDNA metabarcoding to unravel the “missing biomass”^1^ important for fisheries. Even with minimal volumes of six liters of water it is possible to find metabarcode COI of abundant fish species and their preys from the open seas. DNA metabarcoding is a methodology that enables accurate identification of plankton community, in general, and fish species in particular, avoiding the sampling and identification of all fish, fish larvae and eggs present in plankton samples. However, the generation of a reliable identification requires a comprehensive and well-validated reference library^27^. In some regions, DNA is employed for species identification of ichthyoplankton, and there are species-specific markers useful for this purpose in several fish groups of interest such as megrims and hakes^53,54^, cod^55^, horse-mackerel^56^ and others. However, other ichthyoplankton communities are lesser known. Although BOLD system is a promising database for the DNA barcode global research community, and it contains sequences from specimens whose identifications are being refined; the taxonomic coverage and resolution of the COI barcode library on BOLD, and hence the accuracy of identification queries, is still improving^57^. Furthermore, some inconsistencies were found in previous studies developed by our team, with two or more reference sequences from different species that were identical in BOLD, even from different families^11^. On the other hand, although the taxonomic reliability of GenBank has often been questioned^58^, the actual overall data quality is much better than often assumed^59^, still being the most complete database in number of markers and species^27^. Therefore, although Barcode of Life projects are considered a promising tool to species identification, due to the decrease in analysis efforts and costs^60^, DNA-based methodologies are not commonly used and ichthyoplankton still depend on the taxonomic expertise of local pelagic specialists in many regions.

On the other hand, although the number of species detected was small and limited to abundant ones, given the low densities of zooplankton in the open ocean waters, the result is noticeable. It seems that smaller volumes would not be useful to detect zooplankton from open waters with COI metabarcode, since the results of 1.5 L samples were all negative, except for the first sample obtained near the coast. Assaying different water volumes would be recommended to reach an optimal balance between [easy sampling + rapid filtration] and [DNA yield + species capture] in open seas.

Several species found in our African metabarcodes are important for fisheries: the anchovy *Engraulis encrasicolus* is an important fish resource in West Africa, while *Evadne spinifera*, *Clausocalanus furcatus* and the krill *Euphausia* spp. are important in fish diet, the latter being preferred by hake juveniles in African waters^61^. The exception was the Pacific oyster *Magallana gigas,* that is not native to the region. Cited in CABI (2020) not far from the sampling area of West African #3, its larvae are planktonic and can be dispersed by currents^62^.

The number of species captured with the same 6 L water volume was clearly larger in samples taken near the coast, especially in mesotrophic zones, than in those from open waters. In one of the African open sea samples, only one zooplankton species was found (sample West Africa #2, *Clausocalanus furcatus*). This is consistent with the zooplankton density reported from West African waters, that is generally greater near the coast, for example in the mouth of River Gambia (5250 individuals/m^3^), than offshore, frequently with less than 100 individuals/m^3^^39^. Proportionally, in 6 L it would be 0.6, that is, less than one individual. Thus, the low number of species found from open waters in this study is not surprising. In addition, near the coast it is easy to find DNA from sessile species with a short planktonic phase that not enter offshore waters. Short planktonic stages are typical of many mollusks^63^ and reef fish^64^. However, despite the detection of fewer species in the open ocean, the levels of diversity in 6 L water samples were enough to reveal differences between locations, reflecting at least partial differences in species abundance among sites (see clustering analysis in Fig. 5).

Although expectations are that diversity is greater near the Equator and decreases with latitude^65–67^, some insights about trans-equatorial community variation show something different. In ichthyoplankton, the latitudinal pattern of diversity does not exhibit the expected temperate-tropical cline, reflecting instead a decline in low-oxygen zones^9^. Sample WestAfrica#2, where only DNA of *Clausocalanus furcatus* was found, marked an oligotrophic zone of minimum oxygen. Indeed, this confirms the importance of environmental conditions for shaping plankton communities.

Besides zooplankton, COI metabarcode analysis served to detect a variety of species that included many phytoplankton in coastal sites (Supplementary Table 3). Although typically considered a good barcode for animals^28,29,68^, COI has sufficient phylogenetic definition at species level in red algae and phytoplankton. This has been also found in other studies on locations near the coast, including the Baltic Sea^15^, Bay of Biscay ports^16^, species attached to beached litter^69^, and in ballast water^35,70^.

In contrast with coast samples, most non-zooplankton OTUs detected from West African open waters were large vertebrates that are not part of plankton in any life stage (Supplementary Table 3). The samples were taken from RV Polarstern and it is well known that ships attract many large predators that feed on food leftovers and benefit from other species that surround ships^71,72^. Metabarcoding detects only DNA molecules, and expectedly large individuals shed more DNA than small species do^73^. This would explain the dominance of sequences from sharks and cetaceans in the samples obtained from open waters in this study.

Finally, the significant differences found in the taxonomic depth of the assignments between samples can be explained from different geographical coverage of current databases. Reference databases are growing in several regions where zooplankton metabarcoding analysis is conducted^10^, but most of the current studies remark on the necessity to have well-curated databases with a wider taxonomical and geographical coverage^26^. More barcodes would imply a better approximation to the actual number of species in the marine zooplankton assemblage. If these methods, including protocols based on small water volumes, are adequately developed, and independently validated for specific applications, they could be used for several purposes. One could be locating species of interest for fisheries, like preys of commercial fish species or planktonic larvae of such species as we found in West African samples. An inventory of the ichthyoplankton community is essential for understanding how the trophic chain and by extension the whole ecosystem function, as well as for timely prediction of changes due to ichthyoplankton alterations. Which is linked to another possible application, related to the rapid monitoring of the most abundant species detecting large changes in the community.

## Supplementary Information

Supplementary Tables.
